# Changes Introduced in the Open Reading Frame of Bovine Viral Diarrhea Virus During Serial Infection of Pregnant Swine

**DOI:** 10.3389/fmicb.2020.01138

**Published:** 2020-06-10

**Authors:** Thibaud Kuca, Thomas Passler, Benjamin W. Newcomer, John D. Neill, Patricia K. Galik, Kay P. Riddell, Yijing Zhang, Darrell O. Bayles, Paul H. Walz

**Affiliations:** ^1^Department of Clinical Sciences, College of Veterinary Medicine, Auburn University, Auburn, AL, United States; ^2^Ruminant Diseases and Immunology Research Unit, National Animal Disease Center, Agricultural Research Service, United States Department of Agriculture, Ames, IA, United States; ^3^Department of Pathobiology, College of Veterinary Medicine, Auburn University, Auburn, AL, United States; ^4^Infectious Bacterial Diseases Research Unit, National Animal Disease Center, Agricultural Research Service, United States Department of Agriculture, Ames, IA, United States

**Keywords:** bovine viral diarrhea virus, interspecies transmission, open reading frame, persistent infection, pestivirus, RNA virus, swine, viral genetic diversity

## Abstract

Bovine viral diarrhea virus (BVDV) is one of the most economically important viruses of cattle, but this pathogen is also able to infect pigs, camelids, and a wide range of domestic and wild ruminants. BVDV isolates circulating in animal populations are genetically and antigenically highly diverse. Acute BVDV infections in cattle cause the introduction of many substitutions in the viral genome. Serial infection of pregnant sheep with a BVDV-1b isolate of bovine origin was also associated with great numbers of substitutions. To our knowledge, genomic changes arising during BVDV infections in swine have not been investigated. The purpose of this study was to investigate the changes occurring in the open reading frame (ORF) of BVDV during serial infection of pregnant swine with a BVDV isolate of bovine origin. The BVDV-1b isolate AU526 was serially passaged in six pregnant gilts, two of which gave birth to live piglets congenitally infected with BVDV. The complete ORF sequences of 14 BVDV isolates obtained from pregnant gilts and their piglets were determined. Their analysis revealed that serial transmission of AU526 in pregnant swine resulted in many genomic changes. All isolates of porcine origin shared 32 nucleotide and 12 amino acid differences with the virus inoculum AU526. These changes were detected after a single passage in pregnant swine and were conserved during the subsequent five passages. Amino acid changes occurred primarily in genomic regions encoding the BVDV structural proteins E2 and E^*rns*^. These results suggest that BVDV infections in pregnant swine may contribute significantly to the genetic variability of BVDV and lead to the appearance of adaptive changes.

## Introduction

Bovine viral diarrhea virus (BVDV) is one of the most economically important viruses of cattle, but this pathogen is also able to infect pigs, camelids, and a wide range of domestic and wild ruminants, including sheep, goats, and deer (Passler and Walz, 2010). Epidemiological studies indicate that BVDV infections in pigs are relatively common in some major swine-producing countries including China and Brazil (Deng et al., 2012; Gatto et al., 2018). BVDV RNA was detected in 137 of 511 samples (26.8%) obtained from diseased pigs originating from 11 provinces of China (Deng et al., 2012). Acute BVDV infections in pigs typically result in mild or no signs of illness (Stewart et al., 1971). However, pregnant sows infected with BVDV may suffer severe reproductive losses, including early embryonic death, fetal mummification, fetal growth retardation, stillbirth, and abortion (Terpstra and Wensvoort, 1988; Paton and Done, 1994). In a study by Paton and Done (1994), pregnant sows exposed to BVDV gave birth to partially or completely infected litters. Similar to piglets born to sows exposed to classical swine fever virus (CSFV) during pregnancy, piglets congenitally infected with BVDV may remain infected for life (i.e., persistent infection) or only for several weeks (i.e., chronic infection), after which they seroconvert and clear the virus (Van Oirschot, 1979; Paton and Done, 1994). As described in cattle, persistently infected (PI) pigs shed large amounts of virus in nasal secretions, saliva, urine, and semen and may live for more than 2 years (Paton and Done, 1994; Terpstra and Wensvoort, 1997). Indirect transmission from a PI pig to naïve pigs has been demonstrated (Paton and Done, 1994).

Similar to other members of the genus *Pestivirus* in the family *Flaviviridae*, BVDV has a positive-sense, single-stranded RNA genome that contains a single open reading frame (ORF) encoding a large polyprotein, which is processed to yield at least four structural (C, E^*rns*^, E1, and E2) and eight non-structural (N^*pro*^, p7, NS2, NS3, NS4A, NS4B, NS5A, and NS5B) viral proteins (Collett et al., 1988). BVDV isolates circulating in animal populations are genetically and antigenically highly diverse. Two species, *Bovine viral diarrhea virus 1* and *Bovine viral diarrhea virus 2*, were recognized within the genus *Pestivirus*. They have been recently renamed *Pestivirus A* and *Pestivirus B* based on phylogenetic analysis of conserved amino acid sequences (Smith et al., 2017). A minimum of 21 BVDV-1 subgenotypes (1a–1u) and four BVDV-2 subgenotypes (2a–2d) have been reported (Giammarioli et al., 2015; Simmonds et al., 2017; Yesilbag et al., 2017). The accumulation of point mutations caused by the lack of proofreading activity of the BVDV polymerase is thought to be the main driving force for the emergence of new genomic variants (Steinhauer et al., 1992; Sanjuán et al., 2010).

Greater numbers of nucleotide substitutions were shown to be introduced during serial transmission of BVDV in pregnant sheep than in pregnant cattle (Kuca et al., 2018). In that study, multiple host-specific amino acid changes were detected, six of which occurred in the E2 coding region. Amino acid substitutions have also been identified in the E2 coding region during infection of pregnant sheep and goats with BVDV (Gunn et al., 1992; Paton et al., 1997; Bachofen et al., 2013; Passler et al., 2014). Altogether, these results indicate that BVDV infections in pregnant ruminants contribute to the great genetic variability of BVDV.

To our knowledge, viral genomic changes arising during BVDV infections in swine have not been investigated. The purpose of this study was to investigate the changes occurring in the ORF of BVDV in pregnant swine serially infected with a BVDV-1b isolate of bovine origin (AU526). There are many host barriers and defense mechanisms that prevent interspecies transmission of viruses, including receptor specificity and innate and adaptive immune responses (Parrish et al., 2008). Viral genomic changes would likely be needed to circumvent these obstacles, and therefore, many substitutions were anticipated to occur in pregnant swine serially infected with a BVDV isolate of bovine origin. Given the greater phylogenetic distance between cattle and swine (Price et al., 2005), we also hypothesized that serial infection of pregnant swine would result in more viral genomic changes than previously detected in pregnant sheep serially infected with the same BVDV-1b isolate AU526 (Kuca et al., 2018).

## Materials and Methods

### Animals

All procedures involving pigs were approved by the Institutional Animal Care and Use Committee of Auburn University (No. 2015-2706). Seven American Yorkshire-cross pregnant gilts were obtained from the Auburn University Swine Research and Education Center. This facility was populated in 2006 with offspring from specific pathogen-free pigs and is routinely monitored for occurrence of pathogens by serology or necropsy. Clinical or pathological evidence of porcine parvovirus disease, porcine circovirus associated disease, and porcine reproductive and respiratory syndrome had not been detected since the establishment of this herd. Sows and gilts were routinely vaccinated against porcine parvovirus, *Erysipelothrix rhusiopathiae*, and *Leptospira interrogans* serovars *canicola*, *hardjo*, *icterohaemorrhagiae*, and *pomona* (FarrowSure^®^ GOLD, Zoetis, Inc., Kalamazoo, MI, United States).

Prior to inclusion in the study, gilts were confirmed to be pregnant by transabdominal ultrasonography and free of BVDV and antibodies to BVDV by virus isolation (VI) and virus neutralization (VN), respectively. Gilts were transported to the Sugg Laboratory Isolation building at Auburn University and housed in isolation rooms under biosafety level 2 containment conditions. Gilts were visually examined daily for signs of respiratory, gastrointestinal, and reproductive disease during the entire study period. Gilts were identified using the letter P and their order number in the inoculation series. Piglets were identified using the dam’s identification number and a letter referring to the birth order (A, first; B, second; C, third; etc.).

### Cells

Madin-Darby bovine kidney (MDBK) cells were obtained from the American Type Cell Culture Collection (CCL-22^TM^). Cells were confirmed by an independent laboratory to be of bovine origin without *Mycoplasma* or mammalian interspecies contamination (CellCheck^TM^ and STAT-Myco^TM^, IDEXX Laboratories, Inc., Westbrook, ME, United States). Culture medium consisted of minimum essential medium supplemented with Earle’s salts and 10% equine serum, sodium bicarbonate (0.7 μg/ml), and penicillin (100 U/ml).

### Virus Inoculation and Sample Collection

Prior to inoculation, physical and ultrasonographic examinations were performed, and the gilts were confirmed to be pregnant and free from signs of clinical disease. Blood was also collected for VI and VN. Gilts were between 27 and 39 days of gestation at the time of inoculation. The first gilt was inoculated intravenously with approximately 1.0 × 10^6^ 50% tissue culture infective dose (TCID_50_) of BVDV-1b AU526. The non-cytopathic BVDV-1b isolate AU526 had been isolated from the serum of a PI cow, which belonged to a research herd at Auburn University Animal Health Research. Virus inoculum was prepared by passaging the virus stock twice in MDBK cells and then by adding 20 μl of stock solution at a titer of 5.0 × 10^7^ TCID_50_/ml to 980 μl of culture medium. A residual sample of the inoculum was stored at −80°C to estimate the actual received dose by virus titration. Titration of the residual inoculum revealed that the first gilt had received 2.5 × 10^4^ TCID_50_ of AU526.

**FIGURE 1:**
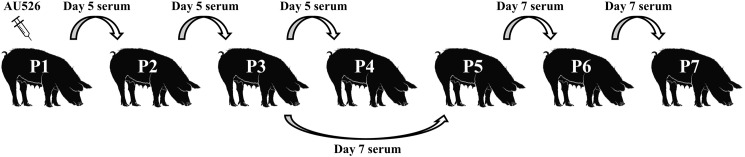
Serial infection of pregnant gilts with BVDV. Gilts were between 27 and 39 days of gestation at the time of inoculation. The first gilt was inoculated intravenously with approximately 1.0 × 10^6^ TCID_50_ of BVDV-1b AU526. The isolate AU526 was serially passaged in pregnant swine by inoculating successively the remaining gilts intravenously with serum obtained from the preceding gilt in the series. The fourth gilt (P4) was inoculated with 1 ml of serum obtained on day 5 from the third gilt (P3) but did not become infected. The fifth gilt (P5) was therefore inoculated with 1 ml of serum obtained on day 7 from P3 to continue the inoculation series.

On days 5 and 7 postinoculation (pi), a physical examination was performed and blood was collected for VI and reverse transcriptase nested PCR (RT-nPCR). The BVDV-1b isolate AU526 was serially passaged in pregnant swine by inoculating successively the remaining gilts intravenously with serum obtained from the preceding gilt in the series (Figure 1). The second, third, and fourth gilts were thus inoculated with 1 ml of serum obtained on day 5 pi from the first, second, and third gilt. Similarly, the fifth, sixth, and seventh gilts were inoculated with 1 ml of serum obtained on day 7 pi from the third, fifth, and sixth gilt, respectively. The fourth gilt (P4) was inoculated with 1 ml of serum obtained on day 5 from the third gilt (P3) but did not become infected. The fifth gilt (P5) was therefore inoculated with 1 ml of serum obtained on day 7 from P3 to continue the inoculation series.

Pregnant gilts were followed to term and housed individually or in pairs following inoculation. Pregnancy viability and BVDV antibody titers were assessed every 28 days by ultrasonography and VN, respectively. Gilts were moved to individual isolation rooms 1 week prior to the anticipated date of farrowing.

Blood was collected from the gilts for VN at the time of parturition. Skin biopsy samples were collected from piglets for antigen-capture enzyme-linked immunosorbent assay (ACE) and reverse transcriptase PCR (RT-PCR). Postmortem examinations were performed on stillborn and deceased piglets. Representative sections of fetal and placental tissues were collected for VI including thymus, spleen, lymph nodes, liver, heart, kidney, lung, brain, gonad, and intestine. Blood was also collected from viable piglets for VI and VN at 21, 42, and 84 days of age.

### Sample Processing

Blood collected in plain and EDTA-containing tubes was processed to yield serum and buffy coat as previously described (Kuca et al., 2018). Buffy coats were immediately used in VI and RT-nPCR procedures, whereas serum samples were stored at −80°C or immediately used in VI, VN, and RT-nPCR procedures. Nasal swabs were placed in tubes containing culture medium, whereas skin biopsy samples were placed in tubes containing phosphate-buffered saline (PBS). Tissue sections were placed in stomacher bags containing 3 ml of culture medium and homogenized for 5 min with a Tekmar Stomacher^®^ laboratory blender (Tekmar Company, Cincinnati, OH, United States).

### Virus Isolation

Serum, buffy coat, nasal swab, and tissue samples were passaged once in MDBK cells for 4 days. An immunoperoxidase monolayer assay (IPMA) was then performed to identify BVDV-positive cells as previously described (Kuca et al., 2018). Two monoclonal antibodies, D89 (VMRD, Inc., Pullman, WA, United States) and 20.10.6 (E. J. Dubovi, Cornell University, Ithaca, NY, United States), were used at a final concentration of 1 and 10 μg/ml, respectively. These antibodies were specific for the BVDV envelope glycoprotein E2 (Vickers and Minocha, 1990; Xue et al., 1997) and the non-structural protein NS3 (Corapi et al., 1990), respectively. Antibody binding was detected using diluted horseradish peroxidase-conjugated rabbit anti-mouse IgG (Jackson ImmunoResearch Laboratories, Inc., West Grove, PA, United States) and aminoethyl carbazole (AEC) substrate (Thermo Fischer Scientific, Inc., Waltham, MA, United States). Each culture plate contained at least one positive and one negative control sample.

### Virus Titration

Initial inoculum and VI-positive serum, buffy coat, and tissue samples were used in virus titration procedures. Multiple 10-fold dilutions of samples (from 10^–1^ up to 10^–7^) were performed to determine viral titers using the statistical method of Reed and Muench (1938) as previously described (Kuca et al., 2018). BVDV-positive cells were identified using the aforementioned IPMA.

### Virus Neutralization

Neutralizing antibody titers to AU526 in serum samples obtained from the gilts and their piglets were determined using a standard VN microtiter assay as previously described (Kuca et al., 2018). All serum samples were assayed in triplicate and antibody titers were defined as the reciprocal of the highest dilution at which two out of three wells were free of staining.

### Antigen-Capture Enzyme-Linked Immunosorbent Assay

The presence of BVDV antigen in skin biopsy samples obtained from the piglets was determined by an independent laboratory using a commercially available ACE kit (BVDV PI X2 Test, IDEXX Laboratories, Inc.) according to the manufacturer’s instructions. Skin biopsy samples were soaked in PBS containing preservative at room temperature for at least 10 min. First, 50 μl of detector antibody was added to each well of a microtiter plate that had been coated with antibodies specific for the BVDV envelope glycoprotein E^*rns*^. Each sample was assayed by then adding 50 μl of processed sample to a well. After incubating at 37°C for 60 min, the plates were washed four times and 100 μl of horseradish peroxidase-conjugated streptavidin was added to each well. The following incubation steps were carried out at room temperature. After incubating for 30 min, the plates were washed four times and 100 μl of 3,3′,5,5′-tetramethylbenzidine (TMB) substrate was added to each well. After incubating for 10 min, the color reaction was stopped by adding 100 μl of stop solution to each well. Each plate contained two negative and two positive control samples. The absorbance values of the samples and controls was determined at a wavelength of 450 nm. The absorbance values of the samples and the mean absorbance value of the positive controls were corrected by subtracting from them the mean absorbance value of the negative controls. Sample to positive ratios were then calculated by dividing the absorbance values of the samples by the mean absorbance value of the positive controls. A sample to positive ratio of 0.3 or greater was considered positive.

### Enzyme-Linked Immunosorbent Assay

The presence of antibodies to porcine circovirus type 2 (PCV2) in serum samples obtained from the gilts on day 0 and on the day of farrowing was determined by an independent laboratory using a commercially available enzyme-linked immunosorbent assay (ELISA) kit (BioCheck, Inc., Scarborough, ME, United States) according to the manufacturer’s instructions. Serum samples were first diluted 1:50 in phosphate buffer containing protein stabilizers and sodium azide preservative (0.2% w/v). All incubation steps were carried out at room temperature. Each sample was assayed by adding 100 μl of diluted sample to a well of a microtiter plate that had been coated with inactivated PCV2. After incubating for 30 min, the plates were washed four times and 100 μl of alkaline phosphatase-conjugated anti-pig antibody was added to each well. After a second round of incubation and washing, 100 μl of prepared substrate reagent containing *p*-nitrophenyl phosphate (pNPP) was added to each well. After incubating for 15 min, the color reaction was stopped by adding 100 μl of stop solution to each well. Each plate contained two negative and two positive control samples. The absorbance values of the samples and controls were determined at a wavelength of 405 nm. The absorbance values of the samples and the mean absorbance value of the positive controls were corrected by subtracting from them the mean absorbance value of the negative controls. Sample to positive ratios were then calculated by dividing the absorbance values of the samples by the mean absorbance value of the positive controls. A sample to positive ratio of 0.5 or greater was considered positive.

### Reverse Transcriptase Nested PCR

Serum and buffy coat samples obtained from the gilts on days 5 and 7 pi were used in RT-nPCR procedures. Viral RNA was extracted using the QIAamp^®^ Viral RNA Mini Kit (Qiagen, Inc., Valencia, CA, United States) according to the manufacturer’s instructions. Viral RNA was stored at −80°C or immediately used.

All steps of the RT-nPCR were performed in a single closed-tube reaction as previously described (Givens et al., 2000). The inner PCR primers HCV 368 and BVD 180 amplified a 213-bp sequence within the first amplicon. The tubes were allowed to dry at room temperature for 2 h before storage. In the second step, 5 μl of viral RNA was added into PCR tubes containing 0.5 μl of Taq polymerase (5 U/μl), 2 μl of dNTPs (10 mM), 1 μl of each outer primer BVD 100 and HCV 368 (5 μM), 10 μl of 10× buffer, 8 μl of MgCl_2_ (25 mM), 1 μl of Triton X-100 (10% stock), 0.25 μl of dithiothreitol (100 mM), 0.25 μl of RNAsin (40 U/μl), 0.5 μl of Moloney murine leukemia virus reverse transcriptase (200 U/μl), and 25.5 μl of RNase-free water. The outer PCR primers BVD 100 and HCV 368 amplified a 290-bp sequence of the 5′ untranslated region of the BVDV genome. First thermal cycling protocol included 45 min of reverse transcription at 37°C and 5 min of polymerase activation and DNA denaturation at 95°C followed by 20 cycles of amplification with denaturation at 94°C for 1 min and primer annealing at 55°C for 1 min and extension at 72°C for 1 min. A final elongation step at 72°C for 10 min completed the initial amplification reaction. In the third step, the tubes were inverted several times to initiate the nested PCR. The tubes were then centrifuged at 14,000 × *g* for 12 s and subjected to 30 thermal cycles at 94°C for 1 min, 55°C for 1 min, and 72°C for 45 s. After a final elongation step at 72°C for 10 min, reactions were maintained at 4°C. PCR products were separated by 1.5% agarose gel electrophoresis. Agarose gels were containing ethidium bromide (0.5 μg/ml) to allow visualization of PCR products using an ultraviolet transilluminator.

### Reverse Transcriptase PCR

The presence of BVDV RNA in skin biopsy samples obtained from the piglets was determined by an independent laboratory using RT-PCR. Viral RNA was extracted using the RNeasy Mini Kit (Qiagen, Inc.) according to the manufacturer’s instructions. The RT-PCR assay was performed on the Applied Biosystems 2720 Thermal Cycler (Thermo Fischer Scientific, Inc.) using SuperScript^TM^ II Reverse Transcriptase (Thermo Fischer Scientific, Inc.), Taq polymerase, and the aforementioned primers (BVD 100, HCV 368, and BVD 180).

### Viral Genome Sequencing

Serum and buffy coat samples that were positive for BVDV by VI but contained less than 10^4^ TCID_50_/ml of virus were passaged once or twice in MDBK cells prior to be used in genome sequencing procedures (Supplementary Table S1). Cell culture growth was limited to two passages to prevent the introduction of artifactual changes in genomic sequences. Infected cells were exposed to a cycle of freezing and thawing to harvest viral isolates that were then stored at −80°C. Genomic sequences of viral isolates were determined as previously described (Kuca et al., 2018). Primers consisting of 20 nucleotides of known sequence with 8 random nucleotides at the 3′ end were utilized to prime cDNA synthesis and to serve as a barcode for identifying each viral library following sequencing. To adapt sequencing to the MiSeq^TM^ platform (Illumina, Inc., San Diego, CA, United States), double-stranded cDNA PCR reactions were transferred to 96-well culture plates and the DNA was size-fractionated and purified using paramagnetic beads (Agencourt^®^ AMPure^®^ XP, Beckman Coulter, Indianapolis, IN, United States) at a DNA-to-bead ratio of 1:0.8. To prepare the DNA for sequencing, the Nextera^TM^ DNA Library Preparation Kit (Illumina, Inc.) was used according to the manufacturer’s instructions. Sequence analysis was performed with the MiSeq^TM^ platform using the MiSeq Reagent kit v2 (Illumina, Inc.) for 2 × 150 base paired-end sequencing. The Lasergene SeqMan NGen software (DNASTAR, Inc., Madison, WI, United States) was used to assemble and edit the genomic sequences. The CodonCode Aligner software (Codoncode Corporation, Centerville, MA, United States) was used to further edit the sequences. Ambiguous nucleotides were assigned when the number of sequencing reads supporting one nucleotide was not greater or equal to 51% of the total number of reads at that position. Numbering of nucleotides started at the ATG initiation codon of the ORF. Each viral isolate was named after the identification number of the animal from which it was isolated. The BVDV-1b isolate AU526 was used as the assembly reference genome. The ORF sequence of this isolate was previously determined using the same sequencing method (Kuca et al., 2018). This isolate was used in that study to inoculate a pregnant heifer and a pregnant ewe. It was then serially passaged in five pregnant heifers and five pregnant ewes by successively inoculating them with serum obtained from the preceding dam in the series. Sequencing was conducted directly on the virus inoculum without any further passage in MDBK cells.

### Accession Numbers

The complete ORF sequences of 14 BVDV-1b isolates were determined in this study and have been deposited in GenBank under accession numbers MH379221 to MH379234. The ORF sequence of the isolate AU526 was previously deposited under accession number MG950344 (Kuca et al., 2018).

### Data Analysis

Pairwise comparisons of viral genomic sequences were performed using MEGA version 10.0.5 (Kumar et al., 2018). Ambiguous nucleotides were not counted as substitutions and therefore were not included in pairwise comparison results.

For all viral isolates obtained from pregnant gilts and their piglets, the ratio of the number of observed differences to the number of expected differences assuming a random distribution of changes across the BVDV genome was calculated for each protein-coding region and viral protein. The number of expected nucleotide and amino acid differences assuming a random distribution of changes was calculated, respectively, using the following formulas:

$$\frac{\mathrm{Total}\,\mathrm{number}\,\mathrm{of}\,\mathrm{observed}\,\mathrm{nucleotide}\,\mathrm{differences}}{\mathrm{Total}\,\mathrm{number}\,\mathrm{of}\,\mathrm{nucleotides}}$$

  ×Number⁢of⁢nucleotides⁢per⁢protein⁢-⁢coding⁢region

$$\frac{\mathrm{Total}\,\mathrm{number}\,\mathrm{of}\,\mathrm{observed}\,\mathrm{amino}\,\mathrm{acid}\,\mathrm{differences}}{\mathrm{Total}\,\mathrm{number}\,\mathrm{of}\,\mathrm{amino}\,\mathrm{acids}}$$

  ×Number⁢of⁢amino⁢acids⁢per⁢viral⁢region

The total number of observed nucleotide and amino acid differences was obtained by comparing the genomic sequence of each viral isolate to AU526. The total number of nucleotides and amino acids was 11,694 and 3,898, respectively. Polyprotein cleavage sites used to calculate the number of nucleotides per protein-coding region and the number of amino acids per viral protein are available in the Supplementary Table S2. Cleavages sites were determined based on previous studies (Rümenapf et al., 1993; Tautz et al., 1997).

### Single Nucleotide Polymorphism Analysis

To determine if the nucleotide changes detected in viral isolates obtained from the gilts and their piglets were initially present as minor variants in the virus inoculum, single nucleotide polymorphism (SNP) analysis was performed on the virus inoculum AU526. BWA-MEM version 0.7.13 (Li, 2013) was used to align sequencing reads to the reference genome, and aligned reads were sorted and indexed with SAMtools version 1.3.1 (Li et al., 2009). Aligned sequencing reads were further processed to remove possible PCR duplicates by applying the Picard Tools MarkDuplicates algorithm^1^. SNPs were detected using both Freebayes version 1.0.2 (Garrison and Marth, 2012) and SNVer version 0.5.3 (Wei et al., 2011) on both the full alignment files and the deduplicated alignment files. Both of the variant callers were run with parameters that optimized outputs for variant calling against a virus reference genome. Raw sequences have been deposited in the NCBI SRA database under accession numbers SRX8229599 to SRX8229602.

### Monoclonal Antibody Binding

A panel of seven monoclonal antibodies (CA1, CA3, CA34, CA82, BZ24, BZ30, and BZ33) that specifically react with the BVDV E2 protein was used to investigate antigenic changes occurring during serial infection of pregnant cattle, sheep, and swine with a BVDV isolate of bovine origin. Monoclonal antibodies were obtained from hybridoma cell cultures as previously described (Bolin et al., 1988). Monoclonal antibody binding patterns were determined using an IPMA in which MDBK cells were infected with viral isolates obtained from the first and last gilts and isolates previously obtained from the first and last heifers and ewes serially inoculated with AU526 (Kuca et al., 2018).

Briefly, culture medium containing 500 TCID_50_ of each viral isolate was added to each of three 0.32-cm^2^ wells of a 96-well culture plate that had been seeded 24 h earlier with MDBK cells. Each culture plate also contained cells infected with AU526 as positive control and mock-infected MDBK cells as negative control. Cultures plates were incubated at 37°C for 3 days and then fixed. Monoclonal antibodies were diluted 1:2 to 1:16 in PBS with bovine albumin (0.01%) and 50 μl of diluted monoclonal preparation were added to each well. Binding procedures were standardized by using the highest dilution of monoclonal antibody that showed no significant loss of reactivity in IPMA against AU526. Incubation at 37°C for 1 h was carried out to allow antibody binding. After washing with PBS containing Tween 20 to remove unbound antibodies, 50 μl of horseradish peroxidase-conjugated rabbit anti-mouse IgG antibody (Jackson ImmunoResearch Laboratories, Inc., West Grove, PA, United States) were added to each well. Following incubation at 37°C for 1 h, another washing was performed and 50 μl of AEC substrate (Thermo Fischer Scientific, Inc., Waltham, MA, United States) were added to each well. Following incubation at room temperature for 15 min, light microscopy was used to determine changes in color. Positive and negative control samples included on each plate were used to compare the results obtained.

## Results

### Clinical, Virological, and Serological Findings

All gilts were confirmed to be free of BVDV and antibodies to BVDV prior to inoculation (Table 1). Serial transmission of BVDV was successfully performed in six pregnant gilts (P1–P3 and P5–P7) as indicated by positive VI and/or RT-nPCR results on day 5 or 7 pi and seroconversion by day 28 pi. The fourth gilt (P4) did not become infected and later delivered nine healthy piglets.

**TABLE 1:** Virological and serological analysis of gilts serially infected with BVDV in early pregnancy.

		**0 dpi**			**5 dpi**		**7 dpi**		**28 dpi**	**56 dpi**		**Day of farrowing**			
**Gilt**	**Fetus^a^**	**VN**	**VI^b^**	**S/P^c^**	**VI^b^**	**RT-nPCR^b^**	**VI^b^**	**RT-nPCR^b^**	**VN**	**VN**	**S/P^c^**	**dpi**	**VN**	**S/P^c^**	**Offspring**
P1	31	<2	−/−	1.374	−/+	+/+	−/+	+/+	512	2,048	NT	83	2,048	1.248	10 live BVDV- piglets/2 mummified piglets
P2	39	<2	−/−	0.647	−/+	+/−	−/+	+/−	128	1,024	NT	78	2,048	0.544	2 live BVDV- piglets/10 mummified piglets/1 stillborn piglet
P3	30	<2	−/−	2.654	−/−	−/−	−/−	+/−	128	1,024	2.986	86	4,096	NT	4 live BVDV- piglets/1 stillborn piglet
P4	39	<2	−/−	2.121	−/−	−/−	−/−	−/−	<2	<2	NT	NA	NT	NT	9 live piglets
P5	27	<2	−/−	2.535	−/+	+/+	−/+	+/+	256	2,048	NT	89	4,096	3.063	6 live BVDV+ piglets/1 mummified piglet
P6	33	<2	−/−	2.946	−/+	+/+	−/+	+/+	512	4,096	NT	84	4,096	2.860	7 live BVDV- piglets/3 mummified piglets
P7	28	<2	−/−	NT	−/+	+/+	−/+	+/+	128	2,048	NT	88	4,096	NT	8 live BVDV+ piglets

*^*a*^Estimated gestational age at the time of inoculation based on artificial insemination date. ^*b*^Results are given for serum samples first, followed by those for buffy coat samples. ^*c*^Anti-PCV2 ELISA antibody sample to positive ratios in serum samples. dpi, day postinoculation; NA, not available; NT, not tested; RT-nPCR, nested reverse transcriptase PCR; VI, virus isolation; VN, virus neutralization; +, positive; −, negative.*

No changes in general condition, vital parameters, and food intake were observed in any of the infected gilts following inoculation. All infected gilts carried the pregnancy to term and delivered 37 live piglets, 16 mummified piglets, and 2 stillborn piglets. The median number of live piglets was 7 (range, 2–10) and the median number of mummified piglets was 2 (range, 0–10). Furthermore, many live piglets had poor viability with 32% of them dying or being euthanized during the first 4 weeks of life. Paired serum samples from all but one infected gilt were analyzed for the presence of antibodies to PCV2. A significant change (≥ 2-fold) in anti-PCV2 antibody concentrations was not detected between day 0 and the day of farrowing.

All piglets born to gilts P5 and P7 were confirmed to be congenitally infected with BVDV by positive ACE and/or RT-PCR results in skin biopsy samples obtained at birth (Table 2). Five piglets born to the fifth gilt (P5A–P5D and P5F) and one piglet born to the seventh gilt (P7A) died or were euthanized within 24 h of birth. Virus could be isolated from tissue samples obtained at necropsy from these six piglets. One piglet born to the fifth gilt (P5E) remained apparently healthy until euthanasia at 182 days of age. A skin biopsy sample obtained from this piglet at 21 days of age tested positive for BVDV by ACE, but another skin sample obtained at 168 days of age tested negative by ACE and RT-PCR. Furthermore, virus could not be isolated from serum or buffy coat samples obtained from this piglet at 21, 42, 84, 126, and 168 days of age or from tissue samples obtained at necropsy. Similarly, two piglets born to the seventh gilt (P7B and P7H) remained apparently healthy until euthanasia at 84 days of age. Skin biopsy samples obtained from these two piglets at 21 days of age tested negative for BVDV by ACE and RT-PCR. Virus could not be isolated from serum or buffy coat samples obtained from these two piglets at 21, 42, and 84 days of age or from tissue samples obtained at necropsy. One piglet from this litter (P7G) died at 26 days of age and virus could not be isolated from tissue samples obtained at necropsy. Conversely, the remaining four piglets born to the seventh gilt (P7C–P7F) were demonstrated to be persistently infected with BVDV by repeated VI from buffy coat samples obtained at 21, 42, and 84 days of age.

**TABLE 2:** Virological analysis of piglets born to gilts infected with BVDV in early pregnancy.

		**Day of birth**			**21 doa**			**42 doa**	**84 doa**	**Day of death or euthanasia**	
		**Skin**		**Tissues^c^**	**Skin**		**BC**	**BC**	**BC**		**Tissues^c^**
**Gilt**	**Fetus^a^**	**ACE^b^**	**RT-PCR^b^**	**VI^b^**	**ACE^b^**	**RT-PCR^b^**	**VI^b^**	**VI^b^**	**VI^b^**	**Doa**	**VI^b^**
P1	31	0/10	0/10	NA	NT	NT	0/10	NT	NT	NT	NT
P2	39	0/3	0/3	0/1	NA	NA	NA	NA	NA	8–9	0/2
P3	30	0/4	0/4	0/1	NT	NT	NT	NT	NT	5–12–13	0/3
P5	27	6/6	6/6	5/5	1/1	0/1	0/1	0/1	0/1	182	0/1
P6	33	0/7	0/7	NA	NT	NT	0/7	NT	NT	NT	NT
P7	28	6/8	8/8	1/1	5/7	3/7	4/7	4/6	4/6	26–84–84	0/3

*^*a*^Estimated gestational age at the time of inoculation based on artificial insemination date. ^*b*^Results are given as the number of positive piglets over the total number of piglets tested. ^*c*^Composite of tissue samples including skin, liver, spleen, thymus, kidney, lung, heart and brain. ACE, antigen-capture ELISA; BC, buffy coat; doa, days of age; NA, not applicable; NT, not tested; RT-PCR, reverse transcriptase PCR; VI, virus isolation.*

### Genomic Sequence Analysis

The complete ORF sequences of 14 BVDV isolates obtained from five acutely infected gilts and nine congenitally infected piglets were determined. Buffy coat samples obtained from pregnant gilts on day 5 or 7 pi and serum or buffy coat samples obtained from piglets at birth or at 21 days of age were used in genome sequencing procedures (Supplementary Table S1). Virus could not be isolated from serum and buffy coat samples obtained from the third gilt (P3). A complete ORF sequence could also not be determined for the piglet P7D. Three complete ORF sequences (P6, P7, and P7A) contained one, three, and four ambiguous nucleotides, respectively.

A median of 41 nucleotide differences (range, 33–45) was detected between the virus inoculum AU526 and viral isolates obtained from acutely infected gilts (Table 3). A median of 49 differences (range, 44–57) was detected between AU526 and isolates obtained from piglets born to these gilts. Most nucleotide changes were transitions with a median transition-to-transversion ratio of 5.8 in isolates from the gilts and of 8.2 in isolates from their piglets. When all isolates obtained from the gilts and their piglets were analyzed, nucleotide changes were most frequently detected in genomic regions encoding non-structural proteins with a median of 10 and 5 differences in the NS3 and NS5B coding regions, respectively. A median of 7 differences was also observed in the E^*rns*^ and E2 coding regions. In all but five isolates there were at least 2.5 times more nucleotide differences in the E^*rns*^ coding region than expected from random distribution (Table 4).

**TABLE 3:** Nucleotide differences between AU526 and viral isolates obtained from pregnant gilts serially infected with BVDV and their congenitally infected piglets.

	**Total**	**Transitions**	**Transversions**	**Ratio**	**N^pro^**	**C**	**E^rns^**	**E1**	**E2**	**p7**	**NS2**	**NS3**	**NS4A**	**NS4B**	**NS5A**	**NS5B**	**% S^a^**	**% NS^b^**
**AU526:Dam**																		
AU526:P1	41	35	6	5.8	1	2	6	3	6	0	4	10	0	2	3	4	41.5	58.5
AU526:P2	42	34	8	4.3	1	2	5	3	7	0	3	11	0	3	2	5	40.5	59.5
AU526:P5	45	40	5	8.0	2	2	7	2	7	0	4	10	0	2	4	5	40.0	60.0
AU526:P6	33	28	5	5.6	1	2	6	2	5	0	2	8	0	1	2	4	45.5	54.5
AU526:P7	35	30	5	6.0	1	2	6	2	7	0	2	8	0	1	2	4	48.6	51.4
Median	41	34	5	5.8	1	2	6	2	7	0	3	10	0	2	2	4	41.5	58.5
Mean	39	33	6	5.9	1	2	6	2	6	0	3	9	0	2	3	4	43.2	56.8
**AU526:Offspring**																		
AU526:P5A	44	36	8	4.5	1	2	7	3	7	0	3	11	0	3	2	5	43.2	56.8
AU526:P5B	47	39	8	4.9	1	2	7	3	10	0	3	11	0	3	2	5	46.8	53.2
AU526:P5C	50	45	5	9.0	2	2	7	2	7	0	6	10	0	4	5	5	36.0	64.0
AU526:P5D	45	37	8	4.6	1	2	7	3	7	0	3	12	0	3	2	5	42.2	57.8
AU526:P5F	48	43	5	8.6	2	2	7	2	7	0	6	10	0	3	4	5	37.5	62.5
AU526:P7A	49	44	5	8.8	2	2	7	3	7	0	5	10	0	3	4	6	38.8	61.2
AU526:P7C	56	50	6	8.3	2	2	7	3	10	0	7	10	0	3	4	8	39.3	60.7
AU526:P7E	57	49	8	6.1	2	2	8	3	11	0	5	11	0	3	4	8	42.1	57.9
AU526:P7F	55	49	6	8.2	2	3	8	4	8	0	5	10	0	4	4	7	41.8	58.2
Median	49	44	6	8.2	2	2	7	3	7	0	5	10	0	3	4	5	41.8	58.2
Mean	50	44	7	7.0	2	2	7	3	8	0	5	11	0	3	3	6	40.9	59.1

*^*a*^Percentage of differences within structural protein-coding regions. ^*b*^Percentage of differences within non-structural protein-coding regions.*

**TABLE 4:** Ratio of the number of observed nucleotide differences per protein-coding region to the number of expected differences assuming a random distribution across the viral genome.

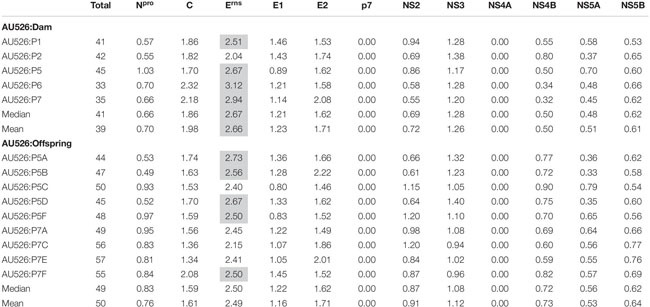

**Ratios greater than 2.5 are shown on a dark gray background.**

Approximately 30% of these nucleotide changes were non-synonymous, resulting in a median of 13 amino acid differences (range, 12–14) between AU526 and isolates obtained from acutely infected gilts. A median of 14 amino acid differences (range, 14–16) was detected between AU526 and isolates obtained from their congenitally infected piglets. When all isolates obtained from the gilts and their piglets were analyzed, amino acid changes were most frequently detected in the E2 coding region with a median of 5 differences (Table 5). A median of 2 differences was also observed in the E^*rns*^ and NS5B coding regions. In all isolates of porcine origin there were at least 2.5 times more amino acid differences in the E2 coding region than expected from random distribution (Table 6). In contrast, no amino acid differences were detected in regions encoding the non-structural proteins N^*pro*^, p7, and NS4A. Similarly, an amino acid change in the C coding region was identified only in the isolate obtained from the piglet P7F (Table 7).

**TABLE 5:** Amino acid differences between AU526 and viral isolates obtained from pregnant gilts serially infected with BVDV and their congenitally infected piglets.

	**Total**	**N^pro^**	**C**	**E^rns^**	**E1**	**E2**	**p7**	**NS2**	**NS3**	**NS4A**	**NS4B**	**NS5A**	**NS5B**	**% S^a^**	**% NS^b^**
**AU526:Dam**															
AU526:P1	13	0	0	2	1	4	0	1	1	0	1	1	2	53.8	46.2
AU526:P2	14	0	0	2	1	5	0	1	1	0	1	1	2	57.1	42.9
AU526:P5	13	0	0	2	1	4	0	2	1	0	0	1	2	53.8	46.2
AU526:P6	12	0	0	2	1	4	0	1	1	0	0	1	2	58.3	41.7
AU526:P7	12	0	0	2	1	4	0	1	1	0	0	1	2	58.3	41.7
Median	13	0	0	2	1	4	0	1	1	0	0	1	2	57.1	42.9
Mean	13	0	0	2	1	4	0	1	1	0	0	1	2	56.3	43.7
**AU526:Offspring**															
AU526:P5A	14	0	0	2	1	5	0	1	1	0	1	1	2	57.1	42.9
AU526:P5B	16	0	0	2	1	7	0	1	1	0	1	1	2	62.5	37.5
AU526:P5C	14	0	0	2	1	4	0	3	1	0	0	1	2	50.0	50.0
AU526:P5D	14	0	0	2	1	5	0	1	1	0	1	1	2	57.1	42.9
AU526:P5F	14	0	0	2	1	4	0	3	1	0	0	1	2	50.0	50.0
AU526:P7A	13	0	0	2	1	4	0	2	1	0	0	1	2	53.8	46.2
AU526:P7C	17	0	0	2	1	6	0	3	1	0	0	1	3	52.9	47.1
AU526:P7E	18	0	0	3	1	8	0	2	1	0	0	1	2	66.7	33.3
AU526:P7F	17	0	1	3	2	5	0	2	1	0	0	1	2	64.7	35.3
Median	14	0	0	2	1	5	0	2	1	0	0	1	2	57.1	42.9
Mean	15	0	0	2	1	5	0	2	1	0	0	1	2	57.2	42.8

*^*a*^Percentage of differences within structural protein-coding regions. ^*b*^Percentage of differences within non-structural protein-coding regions.*

**TABLE 6:** Ratio of the number of observed amino acid differences per viral protein to the number of expected differences assuming a random distribution across the viral genome.

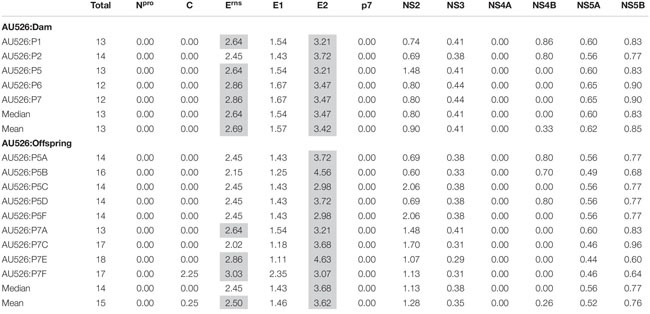

**Ratios greater than 2.5 are shown on a dark gray background*.*

**TABLE 7:** Amino acid changes during serial infection of pregnant gilts with BVDV.

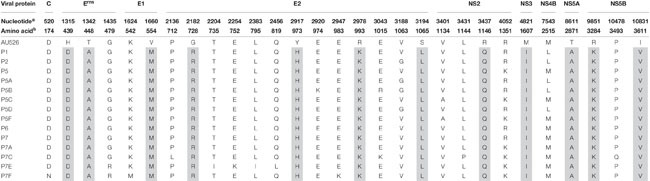

*Conserved changes are shown on a dark gray background. ^*a*^Nucleotides are numbered relative to the ORF of BVDV-1b AU526 (MG950344). ^*b*^Amino acids are numbered relative to the polyprotein of BVDV-1b AU526 (MG950344).*

All isolates of porcine origin shared 32 nucleotide and 12 amino acid differences with the virus inoculum AU526 (Tables 7 and 8). These genomic changes were first detected in the isolate obtained from the first acutely infected gilt (P1) and were subsequently conserved during five serial passages in pregnant swine. One additional nucleotide change (978 T:C) that was identified in the isolate from the fifth gilt (P5) was also detected in the isolates obtained from the last two gilts (P6 and P7) and the piglets born to the fifth and seventh gilts. Conserved nucleotide changes were more prevalent in non-structural protein-coding regions with eight substitutions in the NS3 coding region. In contrast, conserved amino acid changes were found primarily in structural protein-coding regions with four and two changes in the E2 and E^*rns*^ coding regions, respectively. The remaining five conserved amino acid changes occurred in non-structural protein-coding regions with three changes in the NS5B coding region.

**TABLE 8:** Conserved nucleotide changes during serial infection of pregnant gilts with BVDV.

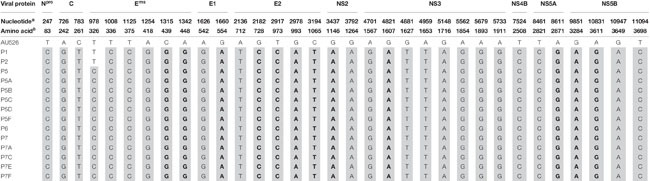

*Conserved changes are shown on a dark gray background and non-synonymous changes are bolded. ^*a*^Nucleotides are numbered relative to the ORF of BVDV-1b AU526 (MG950344). ^*b*^Amino acids are numbered relative to the polyprotein of BVDV-1b AU526.*

Results of all pairwise comparisons as well as the location and type of changes observed between AU526 and viral isolates obtained from acutely infected gilts and their congenitally infected piglets are available in the Supplementary Tables S3–S7. Virus titers in samples obtained from pregnant gilts and their piglets are also available in the Supplementary Tables S8 and S9.

### Comparison With Other BVDV-1 Isolates

To investigate if the amino acid residues affected by conserved changes during serial infection of pregnant swine with BVDV-1b AU526 were also mutated in other BVDV-1 isolates, the complete ORF sequences of 111 BVDV-1 isolates were obtained from GenBank (Supplementary Table S10). There were 17 BVDV-1a, 26 BVDV-1b, 2 BVDV-1c, 5 BVDV-1d, 1 BVDV-1e, 1 BVDV-1h, 2 BVDV-1k, 3 BVDV-1m, 1 BVDV-1n, 1 BVDV-1o, and 2 BVDV-1q. Information regarding the subgenotype was not available for the remaining 50 isolates. Genomic sequences of these 111 isolates were analyzed and the type and the frequency of amino acid residues at positions where conserved changes were detected in this study are available in Table 9.

**TABLE 9:** Type and frequency of amino acid residues at selected positions in ORF sequences of 111 BVDV-1 isolates, 26 of which were BVDV-1b isolates.

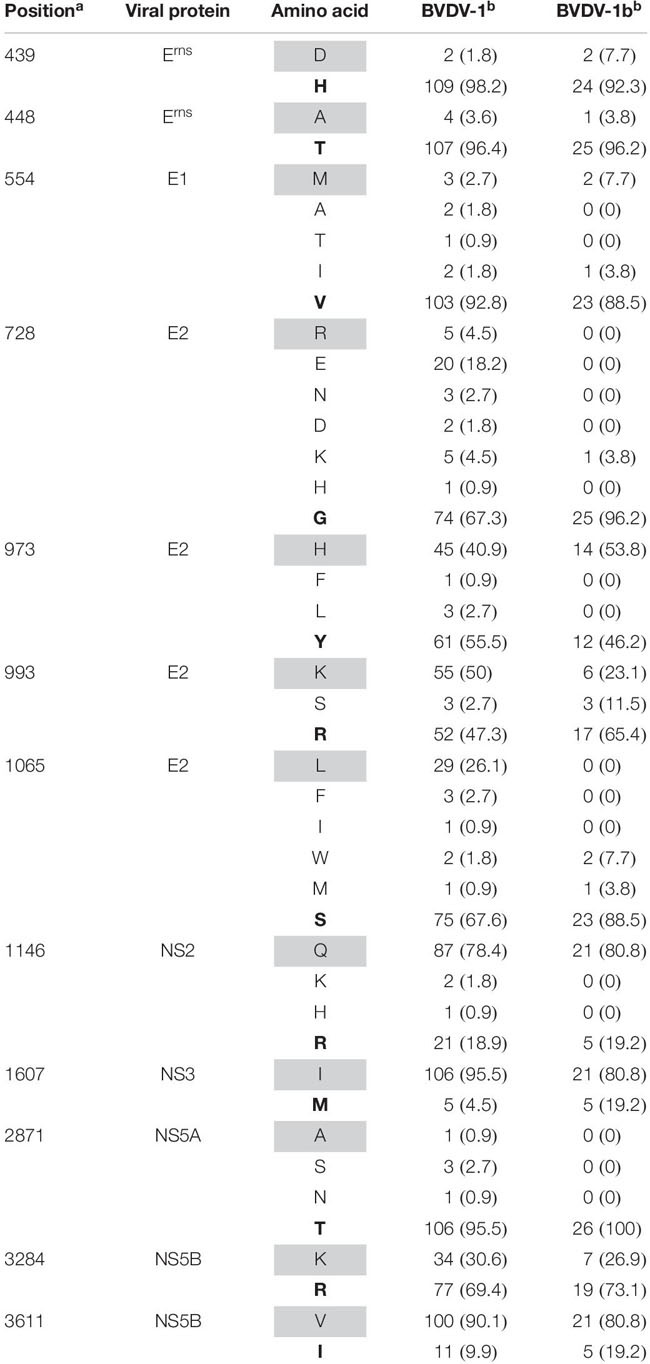

*Mutated amino acid residues that were conserved during serial infection of pregnant gilts with BVDV are shown on a dark gray background. Residues of the polyprotein of AU526 are bolded. ^*a*^Amino acids are numbered relative to the polyprotein of BVDV-1b AU526 (MG950344). ^*b*^Percentages are given in parentheses.*

Amino acid residues at positions 439, 448, 554, and 2871 were highly conserved among all BVDV-1 isolates whereas residues at positions 973, 993, 1146, and 3284 were relatively variable. Furthermore, amino acid residues at positions 1607 and 3611 were less conserved among BVDV-1b isolates compared to the remaining BVDV-1 isolates. Conversely, residues at positions 728 and 1065 were highly conserved among BVDV-1b isolates compared to the remaining BVDV-1 isolates. Less than 10% of BVDV-1 isolates shared residues D439, A448, M554, R728, and A2871 with isolates obtained from pregnant gilts and their congenitally infected piglets. However, most BVDV-1 isolates shared residues Q1146, I1607, and V3611 with these BVDV isolates of porcine origin.

### Single Nucleotide Polymorphism Analysis of the Virus Inoculum AU526

**FIGURE 2:**
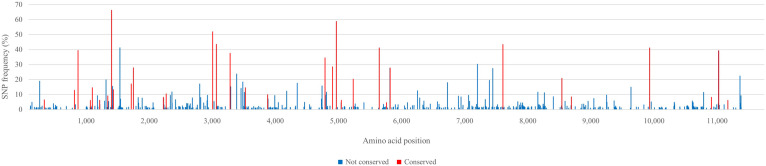
Single nucleotide polymorphism (SNP) frequency plots of the virus inoculum AU526. All SNPs having a frequency above 1% are displayed. Nucleotide changes that were conserved during serial infection of pregnant gilts with BVDV and those that were not conserved are represented as red and blue bars, respectively. Nucleotides are numbered relative to the ORF of BVDV-1b AU526 (MG950344). More detailed information on the results of the SNP analysis can be found in the Supplementary Data Sheet.

Single nucleotide polymorphism (SNP) analysis revealed that the virus population used to inoculate the first pregnant gilt was relatively heterogeneous with 22 SNPs having a frequency above 20% (Figure 2). There were 502 additional SNPs that had frequencies between 1 and 20%. Most of the SNPs that had a frequency above 20% (16 out of 22) were changes that were conserved during serial infection of pregnant gilts with AU526. Of these 16 conserved changes, 9 were synonymous and 7 were non-synonymous. The remaining 17 conserved changes had frequencies between 4 and 20%. More detailed information on the results of the SNP analysis can be found in the Supplementary Data Sheet.

### Monoclonal Antibody Binding Assays

Two porcine isolates (P1 and P7), two bovine isolates (B1 and B4), and two ovine isolates (O1 and O6) were used in monoclonal antibody binding assays (Kuca et al., 2018). The isolate from the last heifer (B6) was not available for analysis and therefore the isolate from the fourth heifer (B4) was used instead. This isolate was selected because there were only six nucleotide differences between these two isolates. One of these differences was located in the E2 coding region but did not result in an amino acid change. All monoclonal antibodies (CA1, CA3, CA34, CA82, BZ24, BZ30, and BZ33) reacted positively and similarly with AU526 and the six aforementioned BVDV isolates obtained from serially infected pregnant heifers, ewes, and gilts.

## Discussion

Pregnant sows exposed to BVDV may give birth to partially or completely infected litters (Paton and Done, 1994; Kulcsar et al., 2001). However, transplacental infection of porcine fetuses with BVDV has not been consistently demonstrated in swine (Stewart et al., 1980; Walz et al., 2004; Araujo Pereira et al., 2018). Differences in strain virulence, inoculum size, route of inoculation, and pregnancy stage may explain these discrepancies. In this study, the BVDV-1b isolate AU526 was serially passaged in six early pregnant gilts and transplacental transmission could be confirmed in two gilts (P5 and P7), which resulted in the birth of ten congenitally infected piglets. Among the piglets born to the last gilt, four piglets (P7C–P7F) were viremic for at least 3 months, whereas virus could not be isolated from three piglets (P7B, P7G, and P7H) that tested positive at birth by ACE and RT-PCR. It is possible that BVDV did not infect all porcine fetuses simultaneously but rather spread from fetus to fetus as previously reported with porcine parvovirus (Bachmann et al., 1975). Fetuses infected later in gestation may have been able to mount an immune response and clear the virus. Fetal infection was likely to have occurred in all gilts as numerous mummified piglets (16) were delivered and approximately a third (32%) of live piglets died within 30 days of birth. Similar findings have been described in sows exposed to BVDV during the second third of gestation (Kulcsar et al., 2001).

Persistent and chronic infections have been reported in piglets congenitally infected with BVDV (Paton and Done, 1994; Terpstra and Wensvoort, 1997). Chronically infected piglets were viremic for several weeks but ultimately seroconverted, which was associated with immediate clearance of the virus from the peripheral blood and delayed clearance from the tissues. Virus clearance has been reported to occur up to 6 months of age (Terpstra and Wensvoort, 1997), and therefore, it is possible that the four piglets that were viremic until 3 months of age would have cleared the virus later in life.

The complete ORF sequences of 14 isolates from five acutely infected gilts and nine congenitally infected piglets were determined. Virus could not be isolated either from serum or buffy coat samples obtained from the third gilt (P3) on days 5 and 7 pi and thus an ORF sequence could not be determined. The serum sample obtained from P3 on day 7 pi was only positive for BVDV by RT-nPCR, which suggested a low-level viremia. Acute infections with a low viral load have also been described in pregnant gilts infected with BVDV-2 (Araujo Pereira et al., 2018).

The BVDV-1b isolate AU526 was utilized in this study because of its ability to establish persistent infection in species other than cattle, including goats and white-tailed deer (Passler et al., 2007, 2014). This isolate had previously been serially passaged in six pregnant heifers and six pregnant ewes using an experimental design similar to the one described in this study (Kuca et al., 2018). In that study, serial transmission of BVDV in pregnant sheep resulted in greater numbers of nucleotide and amino acid substitutions than in pregnant cattle.

Contrary to our initial hypothesis, the number of substitutions observed in viral isolates of porcine origin was similar to the median of 46 nucleotide and 13 amino acid changes observed during serial transmission of AU526 in pregnant sheep (Kuca et al., 2018). In this previous study, smaller numbers of genomic changes were detected in pregnant cattle serially infected with AU526, with a median of 23 nucleotide and 6 amino acid substitutions. Altogether, these results suggest that BVDV infections in pregnant swine may contribute significantly to the genetic variability of BVDV. It is possible that the number of substitutions observed in swine and sheep corresponds to the maximum number of changes that can be tolerated in a heterologous host, regardless of its phylogenetic relatedness to cattle.

Nucleotide substitutions were previously found to occur randomly across the viral genome during serial transmission of BVDV in pregnant cattle and sheep (Kuca et al., 2018). In contrast, there was a bias toward nucleotide substitutions in the E^*rns*^ coding region during serial transmission of BVDV in pregnant swine. Furthermore, amino acid changes were detected primarily in the E2 and E^*rns*^ coding regions. Similar findings were obtained during the establishment of persistent infections in cattle (Neill et al., 2011, 2012). Selection by the immune system may have contributed to the emergence of changes in these regions of the BVDV genome considering that the envelope glycoproteins E2 and E^*rns*^ are the main targets of neutralizing antibodies in acutely infected animals (Weiland et al., 1990; Xue et al., 1990; Hulst and Moormann, 1997).

Twelve amino acid changes detected in the viral isolate obtained from the first infected gilt were found to be conserved during five serial passages in pregnant swine. These changes were also detected in piglets born to these gilts and occurred primarily in the E2 and E^*rns*^ coding regions. One amino acid change (728 G:R) was located in the first domain of the BVDV E2 protein, an Ig-like domain exposed on the virus surface and involved in cell binding (El Omari et al., 2013; Li et al., 2013). Two of these changes (439 H:D and 448 T:A) occurred in the carboxy-terminal membrane anchor region of the E^*rns*^ protein. Mutations affecting this region were demonstrated to dramatically increase the secretion of the E^*rns*^ protein from infected cells (Tews and Meyers, 2007; Burrack et al., 2012). Conserved changes in the E2 and E^*rns*^ coding regions have also been reported in BVDV-1b isolates obtained from alpacas and were shown to increase the ability of these viruses to infect alpaca cells (Neill et al., 2015). It is possible that the amino acid changes detected in this study may have optimized the ability of these viral isolates to infect and establish persistent infection in swine.

Similar to other single stranded RNA viruses, BVDV exists as a viral quasispecies within PI animals (Dow et al., 2015; Ridpath et al., 2015). The term quasispecies refers to a complex population of different but closely related viral genomes (Domingo et al., 2012). Marked differences in size and complexity of viral quasispecies were demonstrated among PI cattle (Dow et al., 2015; Ridpath et al., 2015). The results of the SNP analysis of the virus inoculum AU526 revealed a heterogeneous viral population with 22 SNPs having a frequency above 20%. Most of these SNPs were changes that were conserved during serial infection of pregnant gilts with AU526. These results suggested that these conserved changes resulted from the selection of minor virus variants present in the virus inoculum during serial transmission in pregnant swine.

Ten of the twelve conserved amino acid changes detected in pregnant swine were also found to be conserved during serial transmission of AU526 in pregnant sheep (Kuca et al., 2018). However, these changes were detected after a single passage in pregnant swine, whereas they gradually occurred during serial infection of pregnant sheep (Kuca et al., 2018). These results suggested that conserved changes were more rapidly introduced in a species that was less closely related to cattle than sheep. It is currently unknown, and will be the focus of future experiments, if these changes provided a selective advantage in heterologous species and if they would also have occurred with a pestivirus belonging to a different subgenotype or species.

Up to five amino acid substitutions were detected in the E2 coding region in pregnant swine and sheep serially infected with AU526. However, no changes in binding patterns were detected using a panel of seven monoclonal antibodies raised against the E2 protein of BVDV. These results suggested that serial transmission of AU526 in pregnant cattle, sheep, and swine were not associated with antigenic changes in the E2 protein. It is possible that antigenic changes that occurred in additional epitopes were not detected by the utilized methods.

## Conclusion

Great numbers of genomic changes occurred during serial infection of pregnant swine with a BVDV-1b isolate of bovine origin. All viral isolates obtained from pregnant gilts and their piglets shared 32 nucleotide and 12 amino acid differences with the virus inoculum AU526. These changes were detected after a single passage in pregnant swine and were conserved during the subsequent five passages. Amino acid changes occurred primarily in genomic regions encoding the BVDV structural proteins E2 and E^*rns*^. These results suggest that BVDV infections in pregnant swine may contribute significantly to the genetic variability of BVDV and lead to the appearance of adaptive changes.

## Data Availability Statement

The datasets generated for this study can be found in the NCBI GenBank database under accession numbers MH379221 to MH379234 and in the NCBI SRA database under accession numbers SRX8229599 to SRX8229602.

## Ethics Statement

The animal study was reviewed and approved by Institutional Animal Care and Use Committee of Auburn University (2015-2706).

## Author Contributions

All authors listed have made a substantial, direct and intellectual contribution to the work, and approved it for publication.

## Conflict of Interest

The authors declare that the research was conducted in the absence of any commercial or financial relationships that could be construed as a potential conflict of interest.

## Footnotes

1 https://broadinstitute.github.io/picard/
