# Supplementary material for: Changes Introduced in the Open Reading Frame of Bovine Viral Diarrhea Virus During Serial Infection of Pregnant Swine
Source: Front Microbiol. 2020 Jun 10;11:1138. doi: 10.3389/fmicb.2020.01138 (PMC7298064; doi:10.3389/fmicb.2020.01138)
Supplement: Supplementary file 1 [file Data_Sheet_1.docx]

Supplementary Material

Changes Introduced in the Open Reading Frame of Bovine Viral Diarrhea Virus during Serial Infection of Pregnant Swine

Thibaud Kuca, Thomas Passler^*^, Benjamin W. Newcomer, John D. Neill, Patricia K. Galik, Kay P. Riddell, Yijing Zhang, Darrell O. Bayles and Paul H. Walz

*** Correspondence:**

Thomas Passler

[passlth@auburn.edu](mailto:passlth@auburn.edu)

**Table S1.** Serum and buffy coat samples obtained from pregnant gilts serially infected with BVDV and their congenitally infected piglets that were used in genome sequencing procedures following one or two passages in MDBK cells.

| Dam | Cell passage | Sample | Sampling date | Virus titer (TCID_50_/ml) |
| --- | --- | --- | --- | --- |
| P1 | First | Buffy coat | 5 dpi | 3.5 × 10^4^ |
| P2 | First | Buffy coat | 5 dpi | 3.5 × 10^3^ |
| P5 | Second | Buffy coat | 7 dpi | 3.5 × 10^6^ |
| P6 | First | Buffy coat | 7 dpi | 3.5 × 10^5^ |
| P7 | Second | Buffy coat | 7 dpi | 2.0 × 10^7^ |
|  |  |  |  |  |
| Offspring | Cell passage | Sample | Sampling date | Virus titer (TCID_50_/ml) |
| P5A | First | Serum | Day of birth | 3.5 × 10^5^ |
| P5B | First | Serum | Day of birth | 6.2 × 10^5^ |
| P5C | First | Buffy coat | Day of birth | 3.5 × 10^6^ |
| P5D | First | Serum | Day of birth | 3.5 × 10^4^ |
| P5F | First | Serum | Day of birth | 2.0 × 10^6^ |
| P7A | Second | Serum | Day of birth | 3.5 × 10^6^ |
| P7C | Second | Buffy coat | 21 doa | 6.2 × 10^5^ |
| P7E | Second | Buffy coat | 21 doa | 1.1 × 10^6^ |
| P7F | Second | Buffy coat | 21 doa | 2.0 × 10^6^ |

doa, days of age; dpi, day postinoculation; MDBK, Madin-Darby bovine kidney.

**Table S2.** Polyprotein cleavage sites for AU526 and viral isolates obtained from pregnant gilts serially infected with BVDV and their congenitally infected piglets. Cleavage sites were determined based on previous studies (Rumenapf et al., 1993; Tautz et al., 1997).

| Cleavage site |  | Nucleotide position | Amino acid position |
| --- | --- | --- | --- |
| N^pro^-C |  | 504-505 | 168-169 |
| C-E^rns^ |  | 810-811 | 270-271 |
| E^rns^-E1 |  | 1491-1492 | 497-498 |
| E1-E2 |  | 2076-2077 | 692-693 |
| E2-p7 |  | 3198-3199 | 1066-1067 |
| p7-NS2 |  | 3381-3382 | 1127-1128 |
| NS2-NS3 |  | 4596-4597 | 1532-1533 |
| NS3-NS4A |  | 6816-6817 | 2272-2273 |
| NS4A-NS4B |  | 7008-7009 | 2336-2337 |
| NS4B-NS5A |  | 8049-8050 | 2683-2684 |
| NS5A-NS5B |  | 9537-9538 | 3179-3180 |

**Table S3.** Number of nucleotide differences detected between AU526 and viral isolates obtained from pregnant gilts serially infected with BVDV and their congenitally infected piglets.

|  | AU526 | P1 | P2 | P5 | P5A | P5B | P5C | P5D | P5F | P6 | P7 | P7A | P7C | P7E |
| --- | --- | --- | --- | --- | --- | --- | --- | --- | --- | --- | --- | --- | --- | --- |
| P1 | 41 |  |  |  |  |  |  |  |  |  |  |  |  |  |
| P2 | 42 | 9 |  |  |  |  |  |  |  |  |  |  |  |  |
| P5 | 45 | 22 | 23 |  |  |  |  |  |  |  |  |  |  |  |
| P5A | 44 | 11 | 2 | 23 |  |  |  |  |  |  |  |  |  |  |
| P5B | 47 | 14 | 5 | 26 | 3 |  |  |  |  |  |  |  |  |  |
| P5C | 50 | 27 | 28 | 5 | 28 | 31 |  |  |  |  |  |  |  |  |
| P5D | 45 | 12 | 3 | 24 | 1 | 4 | 29 |  |  |  |  |  |  |  |
| P5F | 48 | 25 | 26 | 3 | 26 | 29 | 2 | 27 |  |  |  |  |  |  |
| P6 | 33 | 9 | 10 | 12 | 10 | 13 | 17 | 11 | 15 |  |  |  |  |  |
| P7 | 35 | 9 | 10 | 11 | 9 | 12 | 16 | 10 | 14 | 2 |  |  |  |  |
| P7A | 49 | 26 | 27 | 4 | 27 | 28 | 5 | 28 | 3 | 16 | 15 |  |  |  |
| P7C | 56 | 33 | 34 | 11 | 34 | 35 | 12 | 35 | 10 | 23 | 22 | 6 |  |  |
| P7E | 57 | 34 | 35 | 12 | 35 | 38 | 13 | 36 | 11 | 24 | 23 | 7 | 15 |  |
| P7F | 55 | 32 | 33 | 10 | 33 | 36 | 11 | 34 | 9 | 22 | 21 | 6 | 13 | 12 |

**Table S4.** Number of amino acid differences detected between AU526 and viral isolates obtained from pregnant gilts serially infected with BVDV and their congenitally infected piglets.

|  | AU526 | P1 | P2 | P5 | P5A | P5B | P5C | P5D | P5F | P6 | P7 | P7A | P7C | P7E |
| --- | --- | --- | --- | --- | --- | --- | --- | --- | --- | --- | --- | --- | --- | --- |
| P1 | 13 |  |  |  |  |  |  |  |  |  |  |  |  |  |
| P2 | 14 | 1 |  |  |  |  |  |  |  |  |  |  |  |  |
| P5 | 13 | 2 | 3 |  |  |  |  |  |  |  |  |  |  |  |
| P5A | 14 | 1 | 0 | 3 |  |  |  |  |  |  |  |  |  |  |
| P5B | 16 | 3 | 2 | 5 | 2 |  |  |  |  |  |  |  |  |  |
| P5C | 14 | 3 | 4 | 1 | 4 | 6 |  |  |  |  |  |  |  |  |
| P5D | 14 | 1 | 0 | 3 | 0 | 2 | 4 |  |  |  |  |  |  |  |
| P5F | 14 | 3 | 4 | 1 | 4 | 6 | 0 | 4 |  |  |  |  |  |  |
| P6 | 12 | 0 | 1 | 1 | 1 | 3 | 2 | 1 | 2 |  |  |  |  |  |
| P7 | 12 | 1 | 2 | 1 | 2 | 4 | 2 | 2 | 2 | 0 |  |  |  |  |
| P7A | 13 | 2 | 3 | 0 | 3 | 3 | 1 | 3 | 1 | 1 | 1 |  |  |  |
| P7C | 17 | 6 | 7 | 4 | 7 | 8 | 5 | 7 | 5 | 5 | 5 | 3 |  |  |
| P7E | 18 | 7 | 8 | 5 | 8 | 10 | 6 | 8 | 6 | 6 | 6 | 5 | 9 |  |
| P7F | 17 | 6 | 7 | 4 | 7 | 9 | 5 | 7 | 5 | 5 | 5 | 4 | 8 | 7 |

**Table S5.** Location and type of nucleotide and amino acid changes detected between AU526 and viral isolates obtained from pregnant gilts serially infected with BVDV. Nonsynonymous nucleotide changes are bolded.

| AU526:Dam | Total | Location and type of nucleotide changes | Total | Location and type of amino acid changes |
| --- | --- | --- | --- | --- |
| AU526:P1 | 41 | 247 T:C, 726 A:G, 783 C:T, 912 G:A, 1008 T:C, 1125 G:A, 1254 A:G, **1315 C:G**, **1342 A:G**, 1626 A:G, **1660 G:A**, 1866 A:G, 2136 A:T, **2182 G:C**, 2526 A:G, **2917 T:C**, **2978 G:A**, **3194 C:T**, **3437 G:A**, 3792 G:A, 4212 T:C, 4230 A:G, 4701 A:G, **4821 G:A**, 4881 G:T, 4959 A:T, 5121 A:G, 5148 G:A, 5562 A:G, 5679 A:G, 5733 A:G, 6588 G:A, 7524 T:C, **7543 A:T**, 8461 T:C, 8541 C:T, **8611 A:G**, **9851 G:A**, **10831 A:G**, 10947 G:A, 11094 T:C | 13 | 439 H:D, 448 T:A, 554 V:M, 728 G:R, 973 Y:H, 993 R:K, 1065 S:L, 1146 R:Q, 1607 M:I, 2515 M:L, 2871 T:A, 3284 R:K, 3611 I:V |
| AU526:P2 | 42 | 247 T:C, 726 A:G, 783 C:T, 1008 T:C, 1125 G:A, 1254 A:G, **1315 C:G**, **1342 A:G**, 1626 A:G, **1660 G:A**, 1866 A:G, 2136 A:T, **2182 G:C**, 2526 A:G, **2917 T:C**, **2978 G:A**, **3188 T:G**, **3194 C:T**, **3437 G:A**, 3792 G:A, 4230 A:G, 4701 A:G, **4821 G:A**, 4881 G:T, 4959 A:T, 5121 A:G, 5148 G:A, 5562 A:G, 5679 A:G, 5733 A:G, 6282 T:C, 6585 T:C, 7524 T:C, **7543 A:T**, 7647 G:A, 8461 T:C, **8611 A:G**, **9851 G:A**, **10831 A:G**, 10947 G:A, 11094 T:C, 11601 A:C | 14 | 439 H:D, 448 T:A, 554 V:M, 728 G:R, 973 Y:H, 993 R:K, 1063 V:G, 1065 S:L, 1146 R:Q, 1607 M:I, 2515 M:L, 2871 T:A, 3284 R:K, 3611 I:V |
| AU526:P5 | 45 | 247 T:C, 345 T:C, 726 A:G, 783 C:T, 840 T:C, 978 T:C, 1008 T:C, 1125 G:A, 1254 A:G, **1315 C:G**, **1342 A:G**, 1626 A:G, **1660 G:A**, 2136 A:T, **2182 G:C**, 2598 T:C, 2682 C:T, **2917 T:C**, **2978 G:A**, **3194 C:T**, **3437 G:A**, 3792 G:A, 3837 A:G, **4052 G:A**, 4653 G:A, 4701 A:G, **4821 G:A**, 4881 G:T, 4959 A:T, 5148 G:A, 5562 A:G, 5679 A:G, 5733 A:G, 6486 G:A, 7524 T:C, 7539 G:A, 8184 A:G, 8461 T:C, 8604 G:A, **8611 A:G**, **9851 G:A**, 10710 G:A, **10831 A:G**, 10947 G:A, 11094 T:C | 13 | 439 H:D, 448 T:A, 554 V:M, 728 G:R, 973 Y:H, 993 R:K, 1065 S:L, 1146 R:Q, 1351 R:K, 1607 M:I, 2871 T:A, 3284 R:K, 3611 I:V |
| AU526:P6 | 33 | 247 T:C, 726 A:G, 783 C:T, 978 T:C, 1008 T:C, 1125 G:A, 1254 A:G, **1315 C:G**, **1342 A:G**, 1626 A:G, **1660 G:A**, 2136 A:T, **2182 G:C**, **2917 T:C**, **2978 G:A**, **3194 C:T**, **3437 G:A**, 3792 G:A, 4701 A:G, **4821 G:A**, 4881 G:T, 4959 A:T, 5148 G:A, 5562 A:G, 5679 A:G, 5733 A:G, 7524 T:C, 7543 A:W^a^, 8461 T:C, **8611 A:G**, **9851 G:A**, **10831 A:G**, 10947 G:A, 11094 T:C | 12 | 439 H:D, 448 T:A, 554 V:M, 728 G:R, 973 Y:H, 993 R:K, 1065 S:L, 1146 R:Q, 1607 M:I, 2871 T:A, 3284 R:K, 3611 I:V |
| AU526:P7 | 35 | 247 T:C, 726 A:G, 783 C:T, 975 C:Y^a^, 978 T:C, 1008 T:C, 1125 G:A, 1254 A:G, **1315 C:G**, **1342 A:G**, 1626 A:G, **1660 G:A**, 2136 A:T, **2182 G:C**, 2526 A:G, 2682 C:T, **2917 T:C**, **2978 G:A**, **3194 C:T**, **3437 G:A**, 3792 G:A, 4230 A:R^a^, 4701 A:G, **4821 G:A**, 4881 G:T, 4959 A:T, 5148 G:A, 5562 A:G, 5679 A:G, 5733 A:G, 7524 T:C, 8461 T:C, **8611 A:G**, **9851 G:A**, 10710 G:R^a^, **10831 A:G**, 10947 G:A, 11094 T:C | 12 | 439 H:D, 448 T:A, 554 V:M, 728 G:R, 973 Y:H, 993 R:K, 1065 S:L, 1146 R:Q, 1607 M:I, 2871 T:A, 3284 R:K, 3611 I:V |

^a^ Ambiguous change not included in the total number of nucleotide changes.

**Table S6.** Location and type of nucleotide and amino acid changes detected between AU526 and viral isolates obtained from piglets born to pregnant gilts serially infected with BVDV. Nonsynonymous nucleotide changes are bolded.

| AU526:Offspring | Total | Location and type of nucleotide changes | Total | Location and type of amino acid changes |
| --- | --- | --- | --- | --- |
| AU526:P5A | 44 | 247 T:C, 726 A:G, 783 C:T, 975 C:T, 978 T:C, 1008 T:C, 1125 G:A, 1254 A:G, **1315 C:G**, **1342 A:G**, 1626 A:G, **1660 G:A**, 1866 A:G, 2136 A:T, **2182 G:C**, 2526 A:G, **2917 T:C**, **2978 G:A**, **3188 T:G**, **3194 C:T**, **3437 G:A**, 3792 G:A, 4230 A:G, 4701 A:G, **4821 G:A**, 4881 G:T, 4959 A:T, 5121 A:G, 5148 G:A, 5562 A:G, 5679 A:G, 5733 A:G, 6282 T:C, 6585 T:C, 7524 T:C, **7543 A:T**, 7647 G:A, 8461 T:C, **8611 A:G**, **9851 G:A**, **10831 A:G**, 10947 G:A, 11094 T:C, 11601 A:C | 14 | 439 H:D, 448 T:A, 554 V:M, 728 G:R, 973 Y:H, 993 R:K, 1063 V:G, 1065 S:L, 1146 R:Q, 1607 M:I, 2515 M:L, 2871 T:A, 3284 R:K, 3611 I:V |
| AU526:P5B | 47 | 247 T:C, 726 A:G, 783 C:T, 975 C:T, 978 T:C, 1008 T:C, 1125 G:A, 1254 A:G, **1315 C:G**, **1342 A:G**, 1626 A:G, **1660 G:A**, 1866 A:G, 2136 A:T, **2182 G:C**, 2526 A:G, **2917 T:C**, **2920 G:A**, **2978 G:A**, **3043 G:A^a^**, **3044 A:G^a^**, **3188 T:G**, **3194 C:T**, **3437 G:A**, 3792 G:A, 4230 A:G, 4701 A:G, **4821 G:A**, 4881 G:T, 4959 A:T, 5121 A:G, 5148 G:A, 5562 A:G, 5679 A:G, 5733 A:G, 6282 T:C, 6585 T:C, 7524 T:C, **7543 A:T**, 7647 G:A, 8461 T:C, **8611 A:G**, **9851 G:A**, **10831 A:G**, 10947 G:A, 11094 T:C, 11601 A:C | 16 | 439 H:D, 448 T:A, 554 V:M, 728 G:R, 973 Y:H, 974 E:K, 993 R:K, 1015 E:R, 1063 V:G, 1065 S:L, 1146 R:Q, 1607 M:I, 2515 M:L, 2871 T:A, 3284 R:K, 3611 I:V |
| AU526:P5C | 50 | 247 T:C, 345 T:C, 726 A:G, 783 C:T, 840 T:C, 978 T:C, 1008 T:C, 1125 G:A, 1254 A:G, **1315 C:G**, **1342 A:G**, 1626 A:G, **1660 G:A**, 2136 A:T, **2182 G:C**, 2598 T:C, 2682 C:T, **2917 T:C**, **2978 G:A**, **3194 C:T**, **3401 T:C**, **3437 G:A**, 3792 G:A, 3837 A:G, **4052 G:A**, 4155 A:G, 4653 G:A, 4701 A:G, **4821 G:A**, 4881 G:T, 4959 A:T, 5148 G:A, 5562 A:G, 5679 A:G, 5733 A:G, 6486 G:A, 7524 T:C, 7539 G:A, 7584 T:C, 7788 T:C, 8181 G:A, 8184 A:G, 8461 T:C, 8604 G:A, **8611 A:G**, **9851 G:A**, 10710 G:A, **10831 A:G**, 10947 G:A, 11094 T:C | 14 | 439 H:D, 448 T:A, 554 V:M, 728 G:R, 973 Y:H, 993 R:K, 1065 S:L, 1134 V:A, 1146 R:Q, 1351 R:K, 1607 M:I, 2871 T:A, 3284 R:K, 3611 I:V |
| AU526:P5D | 45 | 247 T:C, 726 A:G, 783 C:T, 975 C:T, 978 T:C, 1008 T:C, 1125 G:A, 1254 A:G, **1315 C:G**, **1342 A:G**, 1626 A:G, **1660 G:A**, 1866 A:G, 2136 A:T, **2182 G:C**, 2526 A:G, **2917 T:C**, **2978 G:A**, **3188 T:G**, **3194 C:T**, **3437 G:A**, 3792 G:A, 4230 A:G, 4701 A:G, **4821 G:A**, 4881 G:T, 4959 A:T, 5121 A:G, 5148 G:A, 5166 A:G, 5562 A:G, 5679 A:G, 5733 A:G, 6282 T:C, 6585 T:C, 7524 T:C, **7543 A:T**, 7647 G:A, 8461 T:C, **8611 A:G**, **9851 G:A**, **10831 A:G**, 10947 G:A, 11094 T:C, 11601 A:C | 14 | 439 H:D, 448 T:A, 554 V:M, 728 G:R, 973 Y:H, 993 R:K, 1063 V:G, 1065 S:L, 1146 R:Q, 1607 M:I, 2515 M:L, 2871 T:A, 3284 R:K, 3611 I:V |
| AU526:P5F | 48 | 247 T:C, 345 T:C, 726 A:G, 783 C:T, 840 T:C, 978 T:C, 1008 T:C, 1125 G:A, 1254 A:G, **1315 C:G**, **1342 A:G**, 1626 A:G, **1660 G:A**, 2136 A:T, **2182 G:C**, 2598 T:C, 2682 C:T, **2917 T:C**, **2978 G:A**, **3194 C:T**, **3401 T:C**, **3437 G:A**, 3792 G:A, 3837 A:G, **4052 G:A**, 4155 A:G, 4653 G:A, 4701 A:G, **4821 G:A**, 4881 G:T, 4959 A:T, 5148 G:A, 5562 A:G, 5679 A:G, 5733 A:G, 6486 G:A, 7524 T:C, 7539 G:A, 7788 T:C, 8184 A:G, 8461 T:C, 8604 G:A, **8611 A:G**, **9851 G:A**, 10710 G:A, **10831 A:G**, 10947 G:A, 11094 T:C | 14 | 439 H:D, 448 T:A, 554 V:M, 728 G:R, 973 Y:H, 993 R:K, 1065 S:L, 1134 V:A, 1146 R:Q, 1351 R:K, 1607 M:I, 2871 T:A, 3284 R:K, 3611 I:V |
| AU526:P7A | 49 | 247 T:C, 345 T:C, 726 A:G, 783 C:T, 840 T:C, 978 T:C, 1008 T:C, 1125 G:A, 1254 A:G, **1315 C:G**, **1342 A:G**, 1626 A:G, **1660 G:A**, 1695 C:T, 2136 A:T, **2182 G:C**, 2598 T:C, 2682 C:T, 2683 G:R^b^, **2917 T:C**, 2920 G:R^b^, **2978 G:A**, 3043 G:R^b^, **3194 C:T**, **3437 G:A**, 3792 G:A, 3837 A:G, **4052 G:A**, 4155 A:G, 4653 G:A, 4701 A:G, **4821 G:A**, 4881 G:T, 4959 A:T, 5148 G:A, 5562 A:G, 5679 A:G, 5733 A:G, 6486 G:A, 7524 T:C, 7539 G:A, 7788 T:C, 8184 A:G, 8461 T:C, 8604 G:A, **8611 A:G**, 9840 C:M^b^, **9851 G:A**, 10710 G:A, **10831 A:G**, 10947 G:A, 10989 A:G, 11094 T:C | 13 | 439 H:D, 448 T:A, 554 V:M, 728 G:R, 973 Y:H, 993 R:K, 1065 S:L, 1146 R:Q, 1351 R:K, 1607 M:I, 2871 T:A, 3284 R:K, 3611 I:V |
| AU526:P7C | 56 | 247 T:C, 345 T:C, 726 A:G, 783 C:T, 840 T:C, 978 T:C, 1008 T:C, 1125 G:A, 1254 A:G, **1315 C:G**, **1342 A:G**, 1626 A:G, **1660 G:A**, 1695 C:T, **2135 C:T**, 2136 A:T, **2182 G:C**, 2562 C:T, 2598 T:C, 2682 C:T, **2917 T:C**, **2978 G:A**, **3043 G:A**, **3194 C:T**, **3431 T:C**, **3437 G:A**, 3792 G:A, 3837 A:G, **4052 G:A**, 4155 A:G, 4194 T:C, 4653 G:A, 4701 A:G, **4821 G:A**, 4881 G:T, 4959 A:T, 5148 G:A, 5562 A:G, 5679 A:G, 5733 A:G, 6486 G:A, 7524 T:C, 7539 G:A, 7788 T:C, 8184 A:G, 8461 T:C, 8604 G:A, **8611 A:G**, **9851 G:A**, **10478 C:A**, 10710 G:A, **10831 A:G**, 10839 T:C, 10947 G:A, 10989 A:G, 11094 T:C | 17 | 439 H:D, 448 T:A, 554 V:M, 712 P:L, 728 G:R, 973 Y:H, 993 R:K, 1015 E:K, 1065 S:L, 1144 L:P, 1146 R:Q, 1351 R:K, 1607 M:I, 2871 T:A, 3284 R:K, 3493 P:Q, 3611 I:V |
| AU526:P7E | 57 | 247 T:C, 345 T:C, 726 A:G, 783 C:T, 840 T:C, 978 T:C, 1008 T:C, 1125 G:A, 1254 A:G, **1315 C:G**, **1342 A:G**, **1435 G:A**, 1626 A:G, **1660 G:A**, 1695 C:T, 2136 A:T, **2182 G:C**, **2204 C:T**, **2254 G:A**, **2383 C:A**, **2456 A:T**, 2598 T:C, 2682 C:T, **2917 T:C**, **2978 G:A**, **3194 C:T**, **3437 G:A**, 3792 G:A, 3837 A:G, **4052 G:A**, 4155 A:G, 4653 G:A, 4701 A:G, **4821 G:A**, 4881 G:T, 4959 A:T, 5148 G:A, 5562 A:G, 5679 A:G, 5733 A:G, 6178 C:T, 6486 G:A, 7524 T:C, 7539 G:A, 7788 T:C, 8184 A:G, 8461 T:C, 8604 G:A, **8611 A:G**, 9840 C:A, **9851 G:A**, 10464 G:A, 10710 G:A, **10831 A:G**, 10947 G:A, 10989 A:G, 11094 T:C | 18 | 439 H:D, 448 T:A, 479 G:R, 554 V:M, 728 G:R, 735 T:I, 752 E:K, 795 L:I, 819 Q:L, 973 Y:H, 993 R:K, 1065 S:L, 1146 R:Q, 1351 R:K, 1607 M:I, 2871 T:A, 3284 R:K, 3611 I:V |
| AU526:P7F | 55 | 247 T:C, 345 T:C, **520 G:A**, 726 A:G, 783 C:T, 840 T:C, 978 T:C, 1008 T:C, 1125 G:A, 1254 A:G, **1315 C:G**, **1342 A:G**, **1435 G:A**, **1625 A:T**, 1626 A:G, **1660 G:A**, 1695 C:T, 2136 A:T, **2182 G:C**, 2598 T:C, 2682 C:T, **2917 T:C**, **2947 G:A**, **2978 G:A**, **3194 C:T**, **3437 G:A**, 3792 G:A, 3837 A:G, **4052 G:A**, 4155 A:G, 4653 G:A, 4701 A:G, **4821 G:A**, 4881 G:T, 4959 A:T, 5148 G:A, 5562 A:G, 5679 A:G, 5733 A:G, 6486 G:A, 7365 C:T, 7524 T:C, 7539 G:A, 7788 T:C, 8184 A:G, 8461 T:C, 8604 G:A, **8611 A:G**, **9851 G:A**, 10626 A:G, 10710 G:A, **10831 A:G**, 10947 G:A, 10989 A:G, 11094 T:C | 17 | 174 D:N, 439 H:D, 448 T:A, 479 G:R, 542 K:M, 554 V:M, 728 G:R, 973 Y:H, 983 E:K, 993 R:K, 1065 S:L, 1146 R:Q, 1351 R:K, 1607 M:I, 2871 T:A, 3284 R:K, 3611 I:V |

^a^ Nucleotide changes associated with the same amino acid change (1015 E:R).

^b^ Ambiguous change not included in the total number of nucleotide changes.

**Table S7.** Location and type of nucleotide and amino acid changes detected between viral isolates obtained from pregnant gilts serially infected with BVDV and those obtained from their congenitally infected piglets. Nonsynonymous nucleotide changes are bolded.

| Dam:Offspring | Total | Location and type of nucleotide changes | Total | Location and type of amino acid changes |
| --- | --- | --- | --- | --- |
| P5:P5A | 23 | 345 C:T, 840 C:T, 975 C:T, 1866 A:G, 2526 A:G, 2598 C:T, 2682 T:C, **3188 T:G**, 3837 G:A, **4052 A:G**, 4230 A:G, 4653 A:G, 5121 A:G, 6282 T:C, 6486 A:G, 6585 T:C, 7539 A:G, **7543 A:T**, 7647 G:A, 8184 G:A, 8604 A:G, 10710 A:G, 11601 A:C | 3 | 1063 V:G, 1351 K:R, 2515 M:L |
| P5:P5B | 26 | 345 C:T, 840 C:T, 975 C:T, 1866 A:G, 2526 A:G, 2598 C:T, 2682 T:C, **2920 G:A**, **3043 G:A^a^**, **3044 A:G^a^**, **3188 T:G**, 3837 G:A, **4052 A:G**, 4230 A:G, 4653 A:G, 5121 A:G, 6282 T:C, 6486 A:G, 6585 T:C, 7539 A:G, **7543 A:T**, 7647 G:A, 8184 G:A, 8604 A:G, 10710 A:G, 11601 A:C | 5 | 974 E:K, 1015 E:R, 1063 V:G, 1351 K:R, 2515 M:L |
| P5:P5C | 5 | **3401 T:C**, 4155 A:G, 7584 T:C, 7788 T:C, 8181 G:A | 1 | 1134 V:A |
| P5:P5D | 24 | 345 C:T, 840 C:T, 975 C:T, 1866 A:G, 2526 A:G, 2598 C:T, 2682 T:C, **3188 T:G**, 3837 G:A, **4052 A:G**, 4230 A:G, 4653 A:G, 5121 A:G, 5166 A:G, 6282 T:C, 6486 A:G, 6585 T:C, 7539 A:G, **7543 A:T**, 7647 G:A, 8184 G:A, 8604 A:G, 10710 A:G, 11601 A:C | 3 | 1063 V:G, 1351 K:R, 2515 M:L |
| P5:P5F | 3 | **3401 T:C**, 4155 A:G, 7788 T:C | 1 | 1134 V:A |
| P7:P7A | 15 | 345 T:C, 840 T:C, 975 Y:C^b^, 1695 C:T, 2526 G:A, 2598 T:C, 2683 G:R^b^, 2920 G:R^b^, 3043 G:R^b^, 3837 A:G, **4052 G:A**, 4155 A:G, 4230 R:A^b^, 4653 G:A, 6486 G:A, 7539 G:A, 7788 T:C, 8184 A:G, 8604 G:A, 9840 C:M^b^, 10710 R:A^b^, 10989 A:G | 1 | 1351 R:K |
| P7:P7C | 22 | 345 T:C, 840 T:C, 975 Y:C^b^, 1695 C:T, **2135 C:T**, 2526 G:A, 2562 C:T, 2598 T:C, **3043 G:A**, **3431 T:C**, 3837 A:G, **4052 G:A**, 4155 A:G, 4194 T:C, 4230 R:A^b^, 4653 G:A, 6486 G:A, 7539 G:A, 7788 T:C, 8184 A:G, 8604 G:A, **10478 C:A**, 10710 R:A^b^, 10839 T:C, 10989 A:G | 5 | 712 P:L, 1015 E:K, 1144 L:P, 1351 R:K, 3493 P:Q |
| P7:P7E | 23 | 345 T:C, 840 T:C, 975 Y:C^b^, **1435 G:A**, 1695 C:T, **2204 C:T**, **2254 G:A**, **2383 C:A**, **2456 A:T**, 2526 G:A, 2598 T:C, 3837 A:G, **4052 G:A**, 4155 A:G, 4230 R:A^b^, 4653 G:A, 6178 C:T, 6486 G:A, 7539 G:A, 7788 T:C, 8184 A:G, 8604 G:A, 9840 C:A, 10464 G:A, 10710 R:A^b^, 10989 A:G | 6 | 479 G:R, 735 T:I, 752 E:K, 795 L:I, 819 Q:L, 1351 R:K |
| P6:P7F | 21 | 345 T:C, **520 G:A**, 840 T:C, 975 Y:C^b^, **1435 G:A**, **1625 A:T**, 1695 C:T, 2526 G:A, 2598 T:C, **2947 G:A**, 3837 A:G, **4052 G:A**, 4155 A:G, 4230 R:A^b^, 4653 G:A, 6486 G:A, 7365 C:T, 7539 G:A, 7788 T:C, 8184 A:G, 8604 G:A, 10626 A:G, 10710 R:A^b^, 10989 A:G | 5 | 174 D:N, 479 G:R, 542 K:M, 983 E:K, 1351 R:K |

^a^ Nucleotide changes associated with the same amino acid change (1015 E:R).

^b^ Ambiguous change not included in the total number of nucleotide changes.

**Table S8.** Virus titers in passaged buffy coat samples obtained from pregnant gilts serially infected with BVDV.

| Gilt |  | Cell passage | Sample | Sampling date | Virus titer (TCID_50_/ml) |
| --- | --- | --- | --- | --- | --- |
| P1 |  | First | Buffy coat | 5 dpi | 3.5 × 10^4^ |
|  |  |  |  | 7 dpi | 6.2 × 10^3^ |
| P2 |  | First | Buffy coat | 5 dpi | 3.5 × 10^3^ |
|  |  |  |  | 7 dpi | 3.5 × 10^3^ |
| P5 |  | First | Buffy coat | 5 dpi | 6.2 × 10^4^ |
|  |  |  |  | 7 dpi | 6.2 × 10^4^ |
| P6 |  | First | Buffy coat | 5 dpi | 2.0 × 10^6^ |
|  |  |  |  | 7 dpi | 3.5 × 10^5^ |
| P7 |  | First | Buffy coat | 5 dpi | 3.5 × 10^6^ |
|  |  |  |  | 7 dpi | 6.2 × 10^6^ |

dpi, day postinoculation.

**Table S9.** Virus titers in passaged serum or buffy coat samples obtained from piglets born to pregnant gilts serially infected with BVDV.

| Piglet |  | Cell passage | Sample | Sampling date | Virus titer (TCID_50_/ml) |
| --- | --- | --- | --- | --- | --- |
| P5A |  | First | Buffy coat | Day of birth | 3.5 × 10^6^ |
| P5B |  | First | Buffy coat | Day of birth | 6.2 × 10^5^ |
| P5C |  | First | Buffy coat | Day of birth | 3.5 × 10^6^ |
| P5D |  | First | Buffy coat | Day of birth | 2.0 × 10^7^ |
| P5F |  | First | Serum | Day of birth | 2.0 × 10^6^ |
| P7A |  | First | Serum | Day of birth | 3.5 × 10^5^ |
| P7C |  | First | Buffy coat | 21 doa | 2.0 × 10^2^ |
| P7D |  | First | Buffy coat | 21 doa | 3.5 × 10^5^ |
| P7E |  | First | Buffy coat | 21 doa | 3.5 × 10^4^ |
| P7F |  | First | Buffy coat | 21 doa | 2.0 × 10^3^ |

doa, days of age.

**Table S10.** BVDV-1 isolates used for comparison with viral isolates obtained from pregnant gilts serially infected with BVDV and their congenitally infected piglets.

| Isolate | Accession number | Genotype |  | Isolate | Accession number | Genotype |
| --- | --- | --- | --- | --- | --- | --- |
| ILLC | U86599.1 | 1 |  | Singer_Arg | DQ088995.2 | 1a |
| CP7-5A | AF220247.1 | 1 |  | 180 | HQ174292.1 | 1a |
| KE9 | EF101530.1 | 1 |  | 8844 | HQ174293.1 | 1a |
| 08GB44-1 | JQ418633.1 | 1 |  | 6010 | JN380080.1 | 1a |
| 08GB45-2 | JQ418634.1 | 1 |  | No.12_E+ | LC068604.1 | 1a |
| RK13/END- | JX419397.1 | 1 |  | No.12_E- | LC068605.1 | 1a |
| RK13/END+ | JX419398.1 | 1 |  | NADL | M31182.1 | 1a |
| Av69 VEDEVAC | KC695814.1 | 1 |  | SD-1 | M96751.1 | 1a |
| 12F004 | KC963967.1 | 1 |  | Singer Arg | MH133206.1 | 1a |
| BVDV JL-1 | KF501393.1 | 1 |  | BVDV BJ-2013 | MH490942.1 | 1a |
| CC13B | KF772785.1 | 1 |  | BVDV1a-JB | MK509773.1 | 1a |
| GS5 | KJ541471.1 | 1 |  | 8824 | HQ174295.1 | 1b |
| IBSP4ncp | KJ620017.1 | 1 |  | 8830 | HQ174296.1 | 1b |
| GX4 | KJ689448.1 | 1 |  | 6151 | JN380083.1 | 1b |
| USMARC-51998 | KP941581.1 | 1 |  | PJ | JN380088.1 | 1b |
| USMARC-53874 | KP941583.1 | 1 |  | Powder | JN380089.1 | 1b |
| USMARC-53875 | KP941584.1 | 1 |  | 3156 | JN644055.1 | 1b |
| USMARC-55477 | KP941586.1 | 1 |  | 3156 | JN704144.1 | 1b |
| USMARC-55478 | KP941587.1 | 1 |  | 10270 | JX297512.1 | 1b |
| USMARC-55922 | KP941588.1 | 1 |  | Aries | JX297513.1 | 1b |
| USMARC-55923 | KP941589.1 | 1 |  | Columba | JX297514.1 | 1b |
| USMARC-55924 | KP941590.1 | 1 |  | Corona | JX297515.1 | 1b |
| USMARC-55925 | KP941591.1 | 1 |  | Gemini | JX297516.1 | 1b |
| USMARC-55926 | KP941592.1 | 1 |  | Hercules | JX297517.1 | 1b |
| USII-S15 | KU159365.1 | 1 |  | Leo | JX297518.1 | 1b |
| BE/061536/2014 | KU200260.1 | 1 |  | Lyra | JX297519.1 | 1b |
| SLO/2407/2006 | KX577637.1 | 1 |  | Mars | JX297520.1 | 1b |
| ACM/BR/2016 | KX857724.1 | 1 |  | Scorpius | JX297521.1 | 1b |
| SLO/1170/2000 | KX987157.1 | 1 |  | 50 | KF835698.1 | 1b |
| SLO/2416/2002 | KY849592.1 | 1 |  | 50a | KF835699.1 | 1b |
| VE/138cp/05 | LT907991.1 | 1 |  | Egy/Ismailia/2014 | KR029825.1 | 1b |
| VE/138ncp/05 | LT907992.1 | 1 |  | HP-KY-RK13 | KT355592.1 | 1b |
| MA/101/05 | LT968777.1 | 1 |  | HJ-1 | KU756226.1 | 1b |
| GSTZ | MF172980.1 | 1 |  | Y2 | KY964311.1 | 1b |
| XZ01 | MF278651.1 | 1 |  | Osloss | M96687.1 | 1b |
| XZ02 | MF278652.1 | 1 |  | BVDV BJ-2016 | MH490943.1 | 1b |
| SWU-Z6 | MF693403.1 | 1 |  | BVDV1b-JH | MK509774.1 | 1b |
| LV01-2018 | MG923683.1 | 1 |  | KS86-1ncp | AB078950.1 | 1c |
| XC | MH166806.1 | 1 |  | Bega-like | KF896608.1 | 1c |
| Nebraska | MH231153.1 | 1 |  | 10JJ-SKR | KC757383.1 | 1d |
| Ho916 | MH379638.1 | 1 |  | BJ1201 | KT943518.1 | 1d |
| SLO/3301/2014 | MH899941.1 | 1 |  | BJ1305 | KT951840.1 | 1d |
| SLO/33529/2015 | MH899942.1 | 1 |  | BJ1308 | KT951841.1 | 1d |
| SLO/1361/2014 | MH899943.1 | 1 |  | LV01-2018 | MG923683.1 | 1d |
| SLO/28537/2017 | MH899944.1 | 1 |  | Carlito | KP313732.1 | 1e |
| SLO/1883/2013 | MH899945.1 | 1 |  | UM/126/07 | LT631725.1 | 1h |
| 20170226 | MK102095.1 | 1 |  | SuwaNcp | KC853440.1 | 1k |
| CA2006 | MK775204.1 | 1 |  | SuwaCp | KC853441.1 | 1k |
| CP7 | U63479.1 | 1 |  | ZM-95 | AF526381.3 | 1m |
| ILLNC | U86600.1 | 1 |  | SD-15 | KR866116.1 | 1m |
| Nose | AB078951.1 | 1 |  | LN-1 | KT896495 | 1m |
| KS86-1cp | AB078952.1 | 1a |  | Shitara/02/06 | LC089876.1 | 1n |
| NoseE+ | AB558133.1 | 1a |  | IS26/01ncp | LC089875.1 | 1o |
| NoseE- | AB558134.1 | 1a |  | SD0803 | JN400273.1 | 1q |
| Oregon | AF041040.1 | 1a |  | camel-6 | KC695810.1 | 1q |
| NADL | AJ133738.1 | 1a |  |  |  |  |
